# "Post"-pandemic Capitalism: Reform or Transform? Comment on "Ensuring Global Health Equity in a Post-pandemic Economy"

**DOI:** 10.34172/ijhpm.2023.7936

**Published:** 2023-05-06

**Authors:** Howard Waitzkin

**Affiliations:** Health Sciences Center and Department of Sociology, University of New Mexico, Albuquerque, NM, USA

**Keywords:** COVID-19, Pandemic, Capitalism, Capitalist State, Reform, Revolution

## Abstract

This commentary expresses appreciation for Professor Labonté’s work, along with some hopefully constructive suggestions. Professor Labonté’s editorial shows ambivalence about reforms within capitalism. Such reforms remain contradictory and unlikely to prevail. Transformation to post-capitalist political economies is an exciting focus of moving beyond the hurtful effects of capitalism. Can "the state… mitigate capitalism’s inherent inegalitarianism"? Problematically, government resides in the capitalist state, whose main purpose is to protect the capitalist economic system. The state’s contradictory characteristics manifest in inadequate measures to protect health, as during the COVID-19 pandemic. "Social determination," referring to illness-generating structures of power and finance, is replacing "social determinants," referring mainly to demographic variables. Problems warranting attention include: capitalist industrial agriculture causing pandemics through destruction of protective natural habitat, structural racism, sexism and social reproduction, social class structure linked to inequality, and expropriation of nature to accumulate capital. Transformation to post-capitalism involves creative construction of new solidarity economies, while creative destructions block smooth functioning of the capitalist system.

 With great respect for the author, here are some hopefully constructive comments and suggestions.

## Contradictions of Reform Within the Capitalist State

 An underlying ambivalence runs through the editorial^1^: At times, the adverse impacts of capitalism on health seem possible to ameliorate through various reforms. At other times, the structural basis of capitalism, especially the requirement of growth to sustain the accumulation of capital, seems to make impossible the achievement of meaningful health-improving reforms. A single message would focus on the contradictions of reforming the global capitalist system, as well as the importance of imagining and acting on moving beyond capitalism for health.

 For instance, the arguments about “ensuring health equity” and improving inequalities of illness and early death through reforms of capitalism generate skepticism. The paper would benefit by discarding the ambivalence and stating clearly that the health-affirming effects of reforms within capitalism remain fundamentally contradictory and unlikely to be sustained. Therefore, the key effort today involves imagining how our societies can transform concretely to post-capitalist political economic systems. Such a transformation involves revolutionary change, the nature of which has become an exciting focus of people’s struggles to move beyond capitalism and its hurtful effects worldwide.

 “It’s easier to imagine the end of the world than the end of our economic system.” This statement, attributed to Fredric Jameson,^2^ conveys how simple it is to visualize scenarios leading to the end of humanity and other life forms (global warming with rising oceans and hot, uninhabitable land masses, nuclear Armageddon, and so forth). The quote also conveys a vacuum of creative thinking that continues to inhibit transcending global capitalism — a system that benefits an increasingly concentrated fragment of the world’s population (now roughly 0.5%) at the expense of the rest of us.^3^ Yet, how to get from A to B, capitalism to post-capitalism, is the question that we need to answer during this critical period of history, when the destructive forces of this system threaten the survival of human beings and other species.

 Most of us find that it is difficult to imagine a viable path from capitalism to post-capitalism (the ‘TINA’ perspective, that is, “There Is No Alternative”). Because it is hard to imagine a viable path from capitalism to post-capitalism, most people addressing our world’s challenges assume that capitalism will continue to exist. Therefore, we engage in peculiar ways of struggling to improve our most important problems without confronting capitalism, even though we recognize that capitalism generates these problems and continues to make them worse.^4^ Constructing innovative knowledge about a transformation that actually can move beyond capitalism is one purpose of this commentary, as well as many efforts that colleagues and I are pursuing during this dangerous yet hopeful period of world history, as discussed further below.

 Professor Lebonté considers the ways that “the state” can “mitigate capitalism’s inherent inegalitarianism.” A major problem, however, arises from the character of the state in which government resides. The state in which government resides is the capitalist state; it is not a neutral state, let alone a state that aims to benefit people other than the small group of those at the top of the pyramid of wealth and power who control the state. Time and again, political economic realities have confirmed Marx and Engels’s claim that the main role of the capitalist state is to protect the capitalist economic system, or, to use their metaphor, the state is the “executive committee of the bourgeoisie.”^5^ The capitalist state secures the conditions for perpetual capital accumulation. Accordingly, despite their seemingly benevolent impact, the welfare state’s functions pertinent to health, as well as public education, housing, transportation, livable wages, and adequate food supplies, are inherently subject to several political economic contradictions.

 First, the welfare components of the capitalist state remain vulnerable to cutbacks, privatization, and elimination during economic crises, as recently exemplified by the extension of austerity policies to the national health programs of most European countries.^6^ Important public programs of the welfare state predictably constrict or disappear as the capitalist state gears up to address the recurrent crises of capitalism.^7^

 These contradictory characteristics of the capitalist state also have manifested in the introduction of measures that undermine public health systems. As demonstrated during the COVID-19 pandemic, for instance, the ability of public health agencies to implement policies seeking to prevent spread of the infection was compromised by pressures from capital to reopen and resume economic activities that would increase community risk. Simultaneously, these public health agencies often could not overcome barriers to equitable provision of vaccines and medications due to the institutionalized monopoly power of pharmaceutical corporations that protected patent restrictions and profitability (p. 244).^8^

 Second, these welfare functions of the capitalist state contribute to false consciousness and hegemonic beliefs about the state’s beneficent potential to ameliorate the excesses of the system. This ideological impact has been termed the state’s “legitimation function” (p. 244).^8,9^ By providing helpful services including healthcare through a national health program, the state legitimates the continuing inequalities and exploitation inherent in the capitalist system. Some national health programs, such as those in England, Scandinavia, and Canada, have tried to reduce inequalities and exploitation, and the successes of these programs have brought legitimacy as parts of strong welfare states. Yet eventually, with the recurrent crises of capitalism in those countries, cutbacks and privatization have generated wide discontent and reduced the perceived legitimacy of the capitalist system.

## Social Determinants Versus Social Determination

 Increasingly, the concept of “social determination,” referring to the social structures of power and finance that generate ill health and early death, is replacing the concept of “social determinants,” referring to “disparities” in mostly demographic characteristics linked to adverse health outcomes (Table). The paper would benefit by referring to this important conceptual distinction, developed most fruitfully so far in Latin American social medicine (p. 177-198),^8,10^ which holds great importance in envisioning and constructing a “post-COVID-19 economy for health.” Professor Labonté’s own work, for instance on trade agreements and international financial institutions, shows how unlikely it is that reforms in the global capitalist system will happen to the extent that social determination will improve substantially.^11^ More concrete examples from that work could help concretize the analysis.

**Table:** Differences Between Social Determinants and Social Determination

**Social Determinants**	**Social Determination**
Society as sum of individuals.	Society as a totality.
Health–illness as dichotomous states.	Health–illness as a dialectic process.
Change achieves equilibrium; functionalist perspective.	Change results from social contradictions that lead to mass movements and social conflicts.
Variables at individual level of analysis, viewed as risk factors: income, education, job, social cohesion.	Hierarchies of determination, production, and reproduction at a societal level.
Social position generates different exposures and vulnerabilities.	Power relations, accumulation of capital, and discrimination (classism, racism, sexism) create inequality, exploitation, and chronic stress, which lead to illness and early death.
Reforms achieved through “political will” can change SDOH as risk factors. Such changes can occur within the global capitalist system.	Meaningful, lasting improvements in social determination will happen only through societal transformation, including moving beyond the characteristics of global capitalism that generate illness, early death, and fundamental threats to the future of humanity and other forms of life on planet earth.
Example: Individual-level poverty is associated with increasing obesity and diabetes. Interventions focus on changing the eating and exercise habits of poor people.	Example: Obesity and diabetes increase when low-income communities lose their ability to grow and to consume healthy foods through collaborative activities that involve physical labor and mutual aid. Unhealthy foods containing high sugar content are promoted by the capitalist food industry, and healthy foods are more expensive or unavailable due to “food deserts” linked to corporate decisions about profitable investments. Interventions focus on self-sufficiency in collaborative food production, distribution, and consumption at the community level, which reduce profiteering and food insecurity.

Abbreviation: SDOH, social determinants of health.

 To reach a “post-COVID-19 economy for health” implies resolution of recurrent pandemics, so the paper would benefit from some analysis of the origins of such pandemics in capitalist food production and distribution. The author could address structural sources of zoonotic infections causing pandemics in capitalist industrial agriculture, especially destruction of natural habitat and production of meat.^12^ This fundamental cause of all zoonotic epidemics during recent decades receives much less attention than it should.

 Structural racism is intrinsic to racial capitalism, whose successes from the beginning have depended on slavery, genocide, and more recent approaches to racialization that inherently exploit poor and marginalized peoples — those whom Frantz Fanon called “the wretched of the earth.”^13^ The editorial does not refer to racism and, in my view, should. There is no scientific basis to argue that genetically determined race exists, certainly not as an important “variable” in the determination of bad health outcomes. But without doubt racism, through its embodiment among oppressed peoples, does help determine illness and early death. Important recent work on racial capitalism, racialization, and critical race theory calls into question the feasibility of health-affirming reforms within the framework of the capitalist economy.^8^

 Sexism and women’s work in social reproduction is an inherent structural condition that creates and reinforces the exploitation of women within racial capitalism and itself figures importantly in the social determination of health outcomes.^8^ Professor Labonté mentions gender equity in the editorial, but this brief reference could be expanded to include the relationship between social reproduction and social determination of health.

 Although the author refers to inequality and briefly mentions a “billionaire class,” social class does not emerge in the editorial as an important conceptual and practical category. Since Engels referred to the “social murder” of workers in the classic seminal source of social epidemiology, *The Condition of the Working Class in England*, the class structure of capitalist society has emerged as arguably the most fundamental cause in the social determination of ill health and early death.^8^ During the current epoch of grotesque inequality, when a tiny elite control most of the world’s wealth, class structure has become even more important to analyze and change, so in my opinion the editorial should include more reference to that key dimension of capitalism and health.

 The expropriation of nature as an essential requirement for the accumulation of capital has figured as a core observation in ecology, at least since Marx and Engels’s analysis of how the accumulation of capital takes place under capitalism. “Robbery” of raw materials, expropriation of land through enclosures and rent (destroying the prior “commons” that facilitated food production), the “metabolic rift” by which racial capitalism fundamentally shifted agricultural processes and destroyed the soil’s nutrients and carbon absorbing capacity by shifting human wastes to water-borne disposal, the subsequent use of toxic fertilizers and hazardous pollution from industrial agriculture, and the role of military organizations as the principal institutional generators of atmospheric carbon dioxide all figure as parts of capitalism’s destructive expropriation of nature.^14^ As one focus of this editorial, green new deals within the framework of a reformed racial capitalism, especially those that depend on new capitalist technologies, warrant at least some analysis from the standpoint of capitalism’s inherent structural tendencies to destroy nature, with profound effects on health and well-being.

## Revolutionary Transformation

 What is the path toward revolutionary transformation of racial capitalism and its pernicious effects in the social determination of health? Millions of people in local communities around the world actually are changing their lives to move beyond capitalism. The characteristics of this transformation involve actions and inactions that are different from what some of us and traditional teachings have viewed as violent conflict. Key features of the transformation include the implementation of solidarity economies, an expansion of local and regional mutual aid, a transcendence of the “leviathan” that comprises the capitalist state with the construction of communal governance structures, and other creative innovations whose reality has become more feasible as people’s (and especially young people’s) options for survival have become much more limited under late capitalism.^8,15^

 These emerging economic transformations hold important implications for health and wellbeing, as exemplified for instance by the prioritization of “buen vivir” (living well) as a core health policy in some countries and localities of Latin America. Such transformations usually involve grassroots, bottom-up activism, rather than top-down policies initiated by political and economic elites. Communal, democratic decision-making processes specifically seek to avoid the top-down tendencies toward coercive political power that occurred in some versions of “actually existing socialism” such as the Soviet Union under Stalin. In post-capitalist society, the “leviathan state” that protects and legitimates a political economic system based on private accumulation of capital gives way to a new political economic system based on protecting planet earth and the beings that live here.^8,15,16^ This transformative scenario deserves more recognition and serious appraisal in any efforts to construct a “post-COVID-19 economy for health.”

 A transition to post-capitalism is already occurring throughout the world in the creative construction of communal organizations that govern themselves and that act to assure the survival and well-being of their participants. Many examples of such efforts already have emerged.^8,15^ Such widespread efforts contrast with the more publicized turn to right-wing authoritarianism in some localities, as well as militarism such as the conflict between Russia and Ukraine.

 The resulting solidarity political economies, first, find ways to create cheap, small-scale, cooperative, pleasant, comfortable, and health-promoting housing units that require very little money, with collaborative solutions to exploitative rent, debt, taxes, and insurance. Second, communal organizations solve the food problem through local production and distribution of healthy food, achieving independence from capitalist agriculture, and local sovereignty in food production and distribution. To facilitate these actions, local and regional solidarity economies can issue their own currencies with work-time equivalents (such as mutual exchange of work units), offering opportunities similar to those proposed by modern monetary theory within the context of capitalism (as mentioned by Professor Lebonté).^8,15^ The implementation of post-capitalist healthcare and public health occurs mainly within the locally organized solidarity political economies.

 In addition to creative constructions, creative destructions aim to slow down or stop the smooth functioning of the capitalist political economy, as already manifested through many examples.^8,15^ Creative destructions do not take place by obtaining police permits for demonstrations, even large ones, but rather by direct actions that actually slow down or stop the key processes of capitalism. Other creative destructions involve diverting our investments and tax payments into post-capitalist solidarity political economies, with awareness of the predictably favorable impacts on health and healthcare. Through such actions, we can realize the joy of stopping our consent to, and unwitting support for, a system that we know damages our health, well-being, and happiness, and that stifles our ability to give and receive humane, high-quality, and accessible healthcare.

## Ethical issues

 Not applicable.

## Competing interests

 Author declares that he has no competing interests.
